# Self-management of stress urinary incontinence: effectiveness of two treatment programmes focused on pelvic floor muscle training, one booklet and one Internet-based

**DOI:** 10.1080/02813432.2019.1640921

**Published:** 2019-07-18

**Authors:** Kajsa Bokne, Malin Sjöström, Eva Samuelsson

**Affiliations:** a Department of Public Health and Clinical Medicine, Family Medicine, Umeå University, Umeå, Sweden;; b Department of Public Health and Clinical Medicine, Unit of Research, Education and Development – Östersund, Umeå University, Umeå, Sweden

**Keywords:** Stress, urinary incontinence, self-management, e-health

## Abstract

**Objectives:** In a previous study, self-management of stress urinary incontinence (SUI), via an Internet-based programme or a booklet improved symptoms and quality of life. We wanted to evaluate the effectiveness of these programmes when implemented for free use, as well as to characterize the users.

**Design:** Pragmatic prospective cohort study.

**Setting and subjects**: Information about the Internet programme and the booklet was provided at www.tät.nu and by nurse midwives. Both programmes included a three-month pelvic floor muscle training (PFMT) programme. Questionnaires were used at the start and after three months.

**Main outcome measures:** Characteristics of the participants regarding age and education. Reductions in symptom severity was measured using the validated ICIQ-UI SF.

**Results:** 109 women using the booklet, and 166 women using the Internet-based programme responded to the pre-treatment questionnaire. Of these, 53 (48.6%) in the booklet group and 27 (16.3%) in the Internet group responded to the follow-up. The mean age of booklet users was higher, 59.4 years vs. 54.5 years (*p =* .005). The proportion of women with post-secondary education was high, 59% in the booklet group and 67% in the Internet group. The mean reduction in the symptom score was 2.6 points (SD 3.4) in the booklet group, and 3.4 (SD 2.9) in the Internet group. These reductions were significant within both groups, with no difference between the groups, and in the same order of magnitude as in the previous randomised controlled study.

**Conclusion:** Two self-management programmes for SUI, one provided as a booklet and one as an Internet-based programme, also rendered clinically relevant improvements when made freely available.

**KEY POINTS**
Female stress urinary incontinence can be treated using self-management programmes focused on pelvic floor muscle training. This study evaluates the effect of two different programmes, one provided as a booklet and one Internet-based, when made freely available to the public.
•Both programmes rendered clinically relevant improvements, in the same order of magnitude as in the previous randomised controlled study.
•Self-management of stress urinary incontinence should be recommended to women that request treatment.

## Introduction

Stress urinary incontinence (SUI) is a widespread condition, especially among women; between 10% and 39% are affected globally [1]. The general symptom definition of SUI is: ‘*the complaint of any involuntary loss of urine on effort or physical exertion (e.g. sporting activities) or on sneezing or coughing*’ [2]. The etiology of SUI is multifactorial and depends on factors such as age, pregnancy, pelvic floor trauma (usually due to vaginal delivery) [3], obesity and chronic coughing [1]. These factors may affect the pelvic floor and thereby cause SUI [1].

SUI may have a negative impact on quality of life, and some women with SUI report avoidance of leakage-provoking activities such as jogging and jumping [1]. Although SUI is such a widespread condition, less than 50% of affected women seek help [4,5]. In a Swedish study, women reported that talking face-to-face to health care professionals about their condition could be both embarrassing and make them feel exposed [6]. At the same time, SUI may be regarded as a natural part of aging, and not problematic [4].

In a JAMA review, the authors recommend unsupervised pelvic floor muscle training (PFMT) and lifestyle advice as first treatment options for female urinary incontinence [7]. Lifestyle modifications, such as weight loss when overweight, have been shown to improve SUI [8]. The efficacy of pelvic floor muscle training for SUI has been well studied [9–11]. Such training aims to increase the strength and muscle endurance of the pelvic floor [12]. Increased muscle volume gives an enhanced structural support to the pelvic organs [13]. Furthermore, PFMT may improve a person’s ability to coordinate and contract their pelvic floor muscles in conjunction with leakage-provoking events, such as jumping or sneezing [12]. Two out of three women with urinary incontinence achieve improvement or continence after PFMT [9]. PFMT is widely used, but there is no specific consensus on how the training should be provided; supervised by a health provider or self-administrated [7].

E-health programmes focusing on lifestyle advice and PFMT have been shown to be effective both in the short and long term for self-management of SUI [14–17]. In an RCT conducted in 2013 by our research group, 250 women with SUI were randomized to either an Internet-based treatment programme or to a control group that received a booklet, both focused on PFMT. Both groups achieved significant and clinically relevant improvements regarding symptoms, quality of life and number of leakages after three months’ treatment, with no significant difference between the groups [16]. The improvements also persisted, as found in one and two-year follow-ups [17]. Both treatment programmes were made freely available to the public after the study.

RCTs that evaluate new treatments are performed with strict participant inclusion and exclusion criteria. When such treatments are used in clinical practice, the characteristics of the patients may be different, which can affect the treatment outcome. Therefore, is it important to monitor the effectiveness of new treatment programmes after they are implemented.

The aim of this study was to study the characteristics of the users, and to evaluate the effectiveness of the booklet and the Internet-based treatment programmes when they were released for free use.

## Materials and methods

This study is based on two different groups of women. One group of women ordered the booklet from our website or received it from a midwife during an appointment. The other group had signed up for the Internet-based treatment after finding our website without any previous contact with the health care system. Both programmes were available at our website: http://www.econtinence.se, where we also provided information about the study. Primary care midwives working in the regional county were informed about and sent the booklet, but other than that no advertising of the present study was carried out. The treatments were not exactly the same, but both informed users about SUI, gave lifestyle advice, and provided a three-month PFMT programme. The PFMT was designed according to the best available evidence, and contained exercises of escalating difficulty levels, both muscle strengthening and endurance exercises. The users were instructed to perform PFMT three times daily, to train at each level for at least one week, and then move on to the next level. The two programmes were identical to those evaluated in the previous RCT [16] with the exception that users of the Internet programme in this present study did not have any e-mail contact with a urotherapist for support during treatment. The web application for the Internet-based programme was developed on the ACKTUS platform [18] in cooperation with researchers at the Department of Computing Science, Umeå University.

All participants were community-dwelling women actively seeking treatment for their SUI. There were otherwise no specific inclusion or exclusion criteria. Those who responded to the initial questionnaire were included in this pragmatic study.

## Initial questionnaire

All women were asked to fill in an initial questionnaire, on a voluntary basis, before starting treatment. Women in the booklet group answered a paper questionnaire, whereas women in the Internet group filled in an online version. The questionnaires differed slightly between the groups, but both included questions regarding age, education and place of residence. We measured symptom severity with the validated and highly recommended questionnaire the International Consultation on Incontinence Questionnaire – Urinary Incontinence Short Form *(*ICIQ-UI SF) [19,20]. The questionnaire contains three questions: one on the frequency of leakage (never, about once a week or less often, two or three times a week, about once a day, several times a day, all the time); one on the amount of leakage (none, a small amount, a moderate amount, a large amount); and one on the impact of leakage on everyday life (on a scale from not at all to a great deal). It also contains a fourth, non-scored, item used for the assessment of type of incontinence. The answers form a score ranging from 0 to 21. The higher the score, the worse the woman experiences her SUI to be. We considered participants as incontinent if they responded that they had some form of leakage in both the questions ‘How often do you leak urine?’ and ‘How much urine do you usually leak?’ from the ICIQ-UI SF questionnaire.

Answers were considered invalid if all the scored ICIQ-UI SF questions were not answered. The total symptom score was used to further categorise the severity of the incontinence (overall score 1–5 = slight, 6–12 = moderate, 13–18 = severe, 19–21 = very severe) [21].

## Follow-up

Women in the booklet group were given a paper document containing the follow-up questionnaire when answering the initial questionnaire, together with an instruction to answer and send it back by post after three months of treatment. Women using the Internet-based treatment programme were automatically emailed a link to an online follow-up questionnaire three months after registering at the site. Both groups were reminded by e-mail to answer the voluntary follow-up questionnaire, which contained questions on symptom severity (ICIQ-UI SF), frequency of training performed during the last four weeks, and whether other types of SUI treatments had been used during this period of time.

## Statistics

We entered data related to the booklet users manually into SPSS. Data from participants using the Internet-based programme was encrypted and saved on secure servers at the Department for Computing Science, Umeå University. The relevant data for this study was exported as a text file, and converted to SPSS for further calculations.

Baseline characteristics are presented as proportions or as means. For comparison of proportions between groups, chi-square tests were used. Means were compared using *t*-tests.

To identify changes in symptom scores, we calculated the mean difference between the initial and follow-up symptom score of those who were incontinent at the start. For the booklet group, we then used the paired t-test to analyse the significance of the improvement, whereas for the Internet group, we used the Wilcoxon Sign Ranks test due to the small number of participants and lack of normal distribution. For comparison of the outcome between the two groups, we used the independent t-test.

For the analysis of frequency of training, we divided the users into two groups; those who reported that they trained more than three times per week, and those who reported that they trained three times per week or less. We excluded participants who reported that they never trained.

We considered *p*-values of < .05 as statistically significant. SPSS version 24.0 was used for all analyses.

## Results

**Figure 1.**
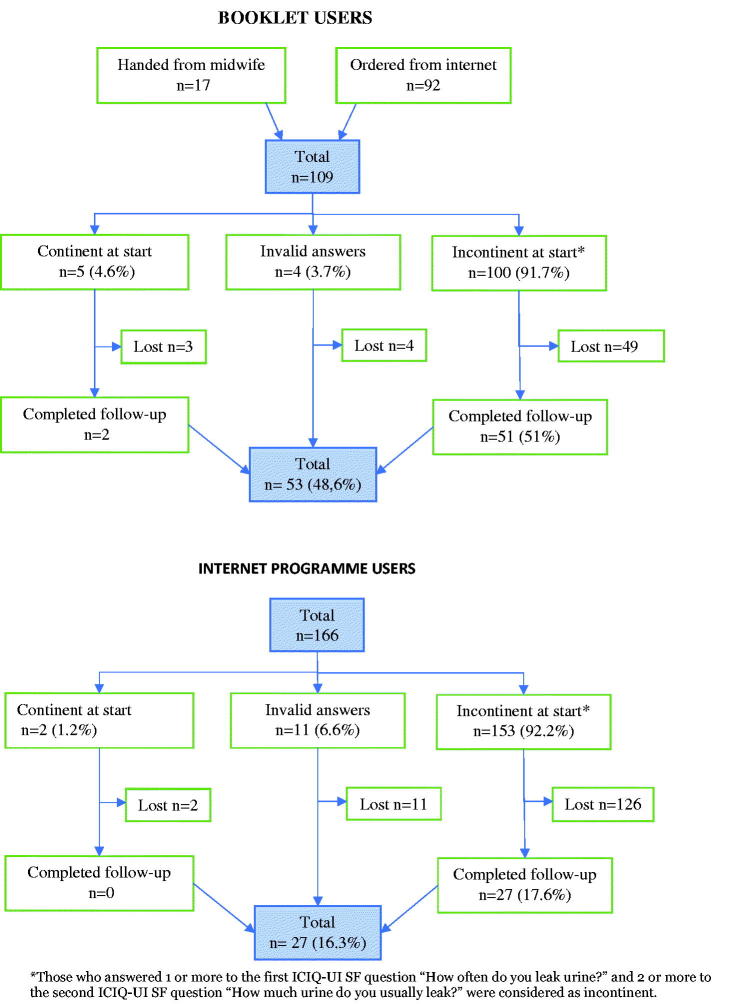
Flow diagram of the booklet and the Internet programme users.

The participants were registered during the period February 2015 to September 2017. The total number of participating women was 109 in the booklet group and 166 in the Internet group. In the booklet group 53 (48.6%) of the participants, and in the Internet group 27 (16.3%) of the participants, responded to the follow-up (*p* < .001) (Figure 1). Women in the booklet group that completed the follow-up tended to be older with a higher level of education than those who did not complete the follow-up, but the differences were not significant (Tables 1 and 2).

**Table 1.** Baseline characteristics of women ordering the **booklet** and those that completed the follow-up.

	Ordered, but did not complete follow-up (*n* = 56)	Ordered and completed follow-up (*n* = 53)	Difference between groups, *p*-value
Age, years – mean (SD)	57.4 (14.3)	61.6 (13.2)	*p* = .12
Highest level of Education – *n* (%)			*p* = .19
Primary school	3 (5.4)	8 (15.1)	
Upper secondary school	20 (36.7)	14 (26.4)	
University	33 (58.9)	31 (58.5)	
Continence – *n* (%)^a^			*p* = .84
Continent	3 (5.8)	2 (3.8)	
Incontinent	49 (94.2)	51 (96.2)	
Slight (3–5)	9 (17.2)	8 (15.1)	
Moderate (6–12)	25 (48.1)	28 (52.8)	
Severe (13–18)	15 (28.8)	14 (26.4)	
Very severe (19–21)	0	1 (1.9)	
Symptom score^b^ – mean (SD)	10.2 (3.8)	10.6 (4.2)	*p* = .97

^a^Those who answered 1 or more to the ICIQ-UI SF question ‘How often do you leak urine?’ and 2 or more to the question ‘How much urine do you usually leak?’ were considered as incontinent. Symptom scores are based on the ICIQ-UI SF questionnaire.

^b^Only women with incontinence included.

**Table 2.** Baseline characteristics of women that started the **Internet programme** and those that completed it.

	Signed up, but did not complete follow-up (*n* = 139)	Signed up and completed follow-up (*n* = 27)	Difference between groups, *p*-value
Age, years – mean (SD)	54.7 (14.5)^a^	53.3 (14.2)	*p* = .64
Highest level of Education – *n* (%)			*p* = .94
Primary school	7 (5.1)	1 (3.7)	
Upper secondary school	38 (27.9)	8 (29.6)	
University	91 (66.9)	18 (66.7)	
Continence – *n* (%)^b^			*p* = .68
Continent	2 (1.6)	0	
Incontinent	126 (98.4)	27 (100)	
Slight (3–5)	13 (10.2)	1 (3.7)	
Moderate (6–12)	73 (57.0)	16 (59.3)	
Severe (13–18)	38 (29.7)	10 (37.0)	
Very severe (19–21)	2 (1.6)	0	
Symptom score– mean (SD)^c^	10.5 (3.9)	11.2 (3.7)	*p* = .38

^a^Based on 134 women.

^b^Those who answered 1 or more to the ICIQ-UI SF question ‘How often do you leak urine?’ and 2 or more to the question ‘How much urine do you usually leak?’ were considered as incontinent. Symptom scores are based on the ICIQ-UI SF questionnaire.

^c^Only women with incontinence included.

### Characteristics of the users

The mean age of the participants was 59.4 years in the booklet group, and 54.5 years in the Internet group (*p* < .001).

The proportion of participants with post-secondary education (university/college degree) was 59% (*n* = 64) in the booklet group (Table 1), and 67% (*n* = 109) in the Internet group (Table 2). The difference between the groups was not significant.

One hundred participants in the booklet group reported incontinence at the start of treatment. Of these, 82 (82%) had moderate or severe incontinence. The mean symptom score in this group was 10.2 (SD 4.0). In the Internet group, 153 participants reported incontinence at the start. Of these, 137 (89.5%) had moderate or severe incontinence. The mean symptom score in this group was 10.6 (SD 3.9). The symptom scores before start of treatment did not differ significantly between the groups.

### Change in symptom scores

The mean reduction in the symptom score was 2.6 (SD 4.3) in the booklet group, and 3.4 (SD 2.9) in the Internet group. The improvement in the symptom score was significant within both groups, with no difference between the groups (Table 3).

**Table 3.** Change in the ICIQ-UI SF symptom score, three months from start.

ICIQ-UI SF Symptom score Mean (SD)	Booklet group^a^*n* = 53	Internet group *n* = 27	Difference, *p*-value, between booklet and internet
Baseline	9.8 (4.6)	11.2 (3.7)	
Follow-up	6.9 (4.3)^b^	7.8 (3.0)	
Change in score	2.6 (3.4)^b^	3.4 (2.9)	
Change in score, *p*-value	<.001	<.001	.08

^a^Only women who filled out the follow-up questionnaire and who had incontinence at start were included.

^b^Based on 48 women.

### Frequency of training

At follow-up, 31 (58.5%) participants in the booklet group and 11 (40.7%) in the Internet group, reported that they trained more than three times per week, *p =* .34.

## Discussion

In this study, we demonstrated that two self-treatment programmes focusing on PFMT, one provided as a booklet and one Internet-based, both rendered significant and clinically relevant improvements for women with SUI when freely available for everyone. The improvements, measured by the recommended and validated ICIQ-UI SF questionnaire, were at the same level as in a previous RCT that had strictly controlled conditions. Women using the booklet were older and completed the three-month PFMT programme to a greater extent than women using the Internet programme.

### Strengths and weaknesses of the study

This study has several strengths. The questionnaire used to measure the severity of incontinence is validated and widely used, which enables us to compare our results with other studies in the same field. We had no technical problems with either the Internet platform or our homepage from which booklets could be ordered during the study. None of the participants reported any problems with understanding either the written information in the booklet or the information given in the Internet-based programme. Also, both programmes have previously been thoroughly tested in an RCT with published results [16].

One weakness of this study is the loss to follow-up. Participants in the booklet group completed the three-month follow-up to a greater extent than participants in the Internet group (48.6% vs. 16.3%). One explanation for the difference between the groups might be that a greater portion of the women in the booklet group initiated treatment after personal contact with health care personnel, whereas all participants in the Internet group initiated treatment on their own accord. However, given that the Internet-based programme was easily accessible to anyone, a big loss is to be expected since those finding it might range from genuinely interested women to those who are just curious. However, the small number of participants in the Internet-group completing the follow-up (*n* = 27) limits the validity of the analyses performed, and also the possibility to evaluate whether the frequency of training correlates with the degree of improvement.

### Characteristics of the users

Some of the participants in the booklet group had had contact with a midwife before the start of treatment, but we never had any contact with the vast majority of our participants, which means that their SUI diagnosis was never confirmed. Although guidelines confirm that SUI can be diagnosed by history alone, we cannot be sure that all participants actually did suffer from SUI. This, of course, is a consequence of releasing the treatment programmes freely to the public. However, since PFMT is also recommended for other common forms of urinary incontinence, such as urgency urinary incontinence and mixed incontinence, this is not a major problem [10]. Furthermore, PFMT is considered a safe form of treatment, side effects are rarely seen, and in those cases that they appear, they are usually reversible [10].

The participants in our study were older than the participants of the original RCT. The mean age in our study was 59.4 years in the booklet group, and 54.5 years in the Internet group, whereas the mean age of participants in the RCT was 48.6 years. However, the RCT was conducted in 2009 to 2010, and this study in 2015 to 2017, and it may be problematic to compare characteristics between studies carried out more than five years apart. During that five-year period, use of the Internet and technical devices such as smartphones and tablets has increased rapidly. From 2010 to 2016, home access to the Internet increased from 85% to 93% among adults in Sweden. Access to smartphones increased from less than 27% to 85% and access to PC tablets or iPads increased from less than 5% to 65% during the same period [22].

Moreover, a large proportion of our participants had university or college education (booklet group 59%, Internet group 67%), and the participants in the RCT tended to be even more highly educated (75.2% had post-secondary education). These proportions are larger than in the general Swedish female population, where the corresponding proportion among women with a mean age of 57 years is 39% [23]. One explanation for this discrepancy might be that highly educated women are known to use the Internet more often to search for health information [24]. However, Henderson et al have previously concluded that educational level does not interfere with the ability to learn how to perform PFMT correctly after short verbal cues [25].

### Improvement of SUI symptoms

The mean score reduction of the validated and highly recommended questionnaire ICIQ-UI SF was 3.4 (SD 2.9) in the Internet group and 2.6 (SD 3.4) in the booklet group when the programmes were freely available. For comparison, the corresponding reductions in the RCT [16] were 3.4 (SD 3.4) and 2.9 (SD 3.1) respectively. Furthermore, the severity of symptoms at start did not differ between participants in this study and participants in the RCT. Moreover, based on data from the RCT, our research group has concluded that an improvement of ≥2.5 at a group level could be considered as clinically relevant as it corresponds to the subjective improvement, ‘a little better,’ according to the validated Patients Global Impression of Improvement form (PGI-I) [26].

The degree of improvement seen in this study is largely equivalent to that seen in other studies based on PFMT. Hirakawa et al [27] conducted a study in which 46 women diagnosed with SUI were randomized to a PFMT programme only, or to PFMT with biofeedback. Both groups had five visits to a physiotherapist during the training. The improvements were 3.4 points (ICIQ-UI SF) in the group using biofeedback and 3.7 points in the group without biofeedback (NS). In another RCT in which 70 women with SUI or MUI (mixed urinary incontinence) underwent a PFMT programme with or without vaginal cones, the reported improvement (ICIQ-UI SF) in the intervention group was 3.3 at three months and 3.9 at six months. All women had two physiotherapist visits and they also received a booklet with instructions [28]. Our research group has previously also demonstrated the efficacy of self-management of SUI through an app-based treatment programme. In this, the app users achieved a mean reduction in the symptom score (ICIQ-UI SF) of 3.9 (95% CI 3.0–4.7), with significant difference from the control group [14].

### Clinical implications and future research

We expected a lower degree of improvement in this study, due to the less strict conditions compared to the RCT, but the reductions in the symptom scores were at the same level of magnitude in both studies. Although the small number of participants is a limitation, we consider that these self-treatment programmes for SUI have been shown to be effective when they are freely available and used in the general population. Both programmes can thus be used as stand-alone treatment for motivated women, and may possibly also serve as a complement to measures recommended within the regular health care system. In addition, our research group has demonstrated the efficacy of mobile app treatment for SUI in another RCT [14], and the app technology brings new possibilities to gather data from users, thus enabling studies of effectiveness in large and non-selected populations.

## Conclusion

This study demonstrated that self-management of stress urinary incontinence provided through the Internet or through a booklet is effective when implemented for free use. The improvements in incontinence symptoms are of the same magnitude as in controlled studies aimed to evaluate the effect of pelvic floor muscle training. Hence, self-treatment should be the first recommendation for women with stress urinary incontinence that are motivated to do non-supervised pelvic floor exercises.
